# Physical Performance and Mental Health in Institutionalized Older Adults: A Multicenter, Cross-Sectional Observational Study

**DOI:** 10.3390/healthcare13243306

**Published:** 2025-12-17

**Authors:** Jorge L. Zambrano, Daniela Zurita-Pinto, Laura Hermo-Rebollido

**Affiliations:** 1 Physical Therapy Department, Faculty of Health Sciences, Universidad Técnica del Norte, Ibarra 100105, Ecuador; dazurita@utn.edu.ec; 2 Psychology Department, Faculty of Science Education, Campus Elviña, Universidade da Coruña, 15006 A Coruña, Spain; l.hermo@udc.es

**Keywords:** physical functioning, depression, older adults, nursing home, anxiety, sleep quality, perceived stress

## Abstract

**Highlights:**

**What are the main findings?**

- Depression and sleep quality are independently and significantly associated with physical functioning in institutionalized older adults, even after adjusting for age and sex.
- In this population, anxiety and perceived stress were not found to have a significant independent association with physical performance scores after statistical adjustment.

**What are the implication of the main findings?**

- Interventions in nursing homes aimed at improving physical performance should prioritize the screening and clinical management of depression and sleep disturbances.
- Targeting mental health factors like depression and poor sleep quality represents a crucial pathway for preserving or enhancing the mobility and functional independence of institutionalized older adults.

**Abstract:**

Aging and institutionalization, associated with functional and psychological decline, justify studying how physical performance is linked to mental health in older adults. **Objectives**: To analyze the relationship between physical performance and anxiety, depression, sleep quality, and perceived stress in institutionalized older adults. **Methods**: Multicenter, observational, cross-sectional study in eight nursing homes (*N* = 105; ≥65 years, M = 80.78 SD ± 7.91). Instruments: SPPB, HADS, PSQI, PSS-10. Descriptives and exploratory bivariate tests. Primary analysis: single multivariable linear regression with SPPB as outcome and HADS-A, HADS-D, PSQI, PSS-10 entered simultaneously, adjusted for age and sex. Robustness: GLM with robust SEs, influence sensitivity excluding Cook’s D > 4/n or leverage > 2 p/n, and a proportional-odds model for SPPB. All statistical tests were two-sided, with α set at 0.05. **Results**: Mean SPPB was 6.94 ± 3.17; 77.1% of participants showed poor physical performance. Bivariate: All mental health constructs showed significant associations with physical performance. Multivariable model: adjusted *R*^2^ = 0.198; *F* (6,98) = 5.28, *p* < 0.001. Depression *B* = −0.230 (95% CI −0.398 to −0.061), *p* = 0.008; sleep quality (higher = worse) *B* = −0.187 (95% CI −0.351 to −0.024), *p* = 0.025; age *B* = −0.087 (95% CI −0.158 to −0.017), *p* = 0.016. Anxiety showed a positive adjusted association *B* = +0.224 (95% CI 0.038 to 0.410), *p* = 0.019 (consistent with suppression); perceived stress *B* = −0.062, *p* = 0.275; sex *B* = −0.144, *p* = 0.812. Robust SEs left inferences unchanged. Influence sensitivity (*n* = 97) preserved directions with the PSQI association attenuating to non-significance. Ordinal results were directionally consistent. **Conclusions**: After adjusting for age and sex, depression and sleep quality independently relate to physical function, while age is inversely associated; anxiety and perceived stress show no independent effects.

## 1. Introduction

Aging is a process of physical, mental, and social transformation that, as age advances, leads to an increase in the prevalence of chronic diseases, functional limitations, and difficulties in performing activities of daily living [1]. It is also associated with a deterioration in mental health and a higher incidence of disorders such as depression and anxiety [2].

This situation becomes even more relevant when considering the rapid growth of the global population of older adults (people over 64 years of age), which is projected to exceed 16% of the total population by 2030 [3]. In Ecuador, figures from the National Institute of Statistics and Census confirm this upward trend [4].

A specifically vulnerable growing population is that of institutionalized older adults. The institutionalization of older adults, and particularly their admission to long-term care homes (nursing homes), has a negative impact on their physical and mental health. Institutionalized older adults are at greater risk of functional dependence and falls due to physical and cognitive decline, added to a lack of environmental stimuli that contributes to cognitive impairment and depression [5].

For this reason, attention to the physical health of older adults is crucial for their overall well-being. Consequently, regular physical activity is essential to maintain functional fitness, mitigate sarcopenia, and reduce the risk of falls [5,6]. These benefits translate into a better quality of life and a more positive perception of aging [7]. In addition, regular exercise contributes to mental health by reducing symptoms of depression and anxiety [6]. However, the prevalence of depression, neurocognitive disorders, and anxiety continues to increase, especially among older adults with multiple comorbidities [2], as well as the deterioration of physical health among people in this age group, as various studies confirm that most older people do not meet the minimum levels of physical activity necessary to maintain health [8,9]. A favorable perception of aging and social support strengthens mental health and quality of life [7], especially when considering that the comprehensive coordination of physical, mental, and social services is essential for the well-being of this population [2].

Several studies have linked poor physical performance—strength, balance, and mobility—with greater dependency and a high prevalence of anxiety, depression, and sleep disturbances [1,10]. However, what remains insufficiently established is which mental-health domains retain independent associations with physical performance when modelled simultaneously—a distinction that is clinically relevant, given that depression, anxiety, sleep quality, and perceived stress often overlap and can inflate bivariate signals. Therefore, to address this limitation, we estimate the concurrent effects of these four domains in a single multivariable framework (adjusted for age and sex) and corroborate findings with robustness tests, thus moving beyond replication toward decision-relevant inference for long-term care.

Although our study is conducted in institutionalized older adults in the Ibarra canton, its primary contribution is this analytical clarification, as this can inform the design of interventions to promote autonomy and emotional well-being and to reduce care costs in this group [11]. In this sense, providing such practice-oriented estimates is therefore both clinically and socially pertinent.

In summary, and in the context of an accelerated demographic transition, identifying the factors that modulate quality of life in institutional settings is a public health priority [12]. Evidence from the literature indicates that physical performance and mental health mutually influence each other and determine the functionality, autonomy, and psychosocial well-being of older adults [13].

### 1.1. Objective

To explore the relationship between physical performance and anxiety and depression, sleep quality, and perceived stress in institutionalized older adults using a single multivariable model adjusted for age and sex.

### 1.2. Hypothesis

**H_0_:**  *In a single multivariable model adjusted for age and sex, there are no independent associations between physical performance and each mental-health domain—anxiety, depression, sleep quality, and perceived stress—in institutionalized older adults.*

**H_1_:**  *In a single multivariable model adjusted for age and sex, physical performance is independently associated with the mental-health domains—anxiety, depression, sleep quality, and perceived stress—in institutionalized older adults.*

## 2. Materials and Methods

### 2.1. Study Design and Ethical Considerations

A cross-sectional, observational, multicenter study was conducted with older adults from eight nursing homes in the Ibarra Canton of Ecuador from March to April 2025.

The study was conducted according to the Declaration of Helsinki and Ecuadorian ethical regulations, and approval was obtained from the governing board of the institutional academic unit (Faculty of Health Sciences belonging to Universidad Técnica del Norte) (Approval ID: UTN-CI-2024-0271-R, 16 October 2024).

Written authorization was obtained from the eight nursing homes, then the research protocol was shared with all older adults and/or caregivers, and their written informed consent was obtained, emphasizing their voluntary participation and confidentiality in data processing.

All data was anonymized (an alphanumeric code was assigned to each participant to ensure confidentiality) and stored on encrypted, password-protected servers, accessible only to study staff.

Reporting guideline: This manuscript adheres to the STROBE Statement—Checklist for cross-sectional studies to enhance transparency and scientific rigor in methods and results reporting.

### 2.2. Participants

The study included older adults from eight public nursing homes in the Ibarra canton, selected by convenience sampling:

1. Santa Luisa de Marillac Nursing Home.
2. Nueva Vida Comprehensive Aid Foundation.
3. Juan Pablo II Nursing Home.
4. La Casa Grande Nursing Home.
5. Santa María Nursing Home.
6. FISMEDICAL Nursing Home.
7. León Rúales Residential Nursing Home.
8. CEDIAAM Nursing Home.

### 2.3. Eligibility Criteria

Individuals aged ≥65 years who had been residing in one of the eight nursing homes in Ibarra for at least one month, who were cardiorespiratory and neurologically stable according to vital parameters (no acute episodes in the last four weeks), who understood and communicated in Spanish with the research team, and who provided their own or their legal representative’s informed consent were included. Those who presented severe cognitive impairment or delirium (Mini-Mental State Score < 11), severe visual, hearing, or motor deficits that prevented evaluation, recent acute illness that limited mobility, life expectancy of less than three months, or refusal/inability to sign consent were excluded.

### 2.4. Sample Size and Precision

We aimed to recruit all eligible residents across the eight nursing homes during the predefined 8-week window (March–April 2025), yielding *N* = 105. Although no a priori power calculation was performed, we provide a post hoc precision statement for the primary contrasts. Given the observed SPPB variability (*SD* ≈ 3.17) and the group sizes for sleep quality (27 good vs. 78 poor), the study has reasonable precision to detect differences around ~2 SPPB points at α = 0.05 (two-sided). The adjusted effect estimates and their 95% CIs reported in the Results fall within this expected precision range. We interpret all findings with caution due to subgroup sizes.

### 2.5. Variables and Measurement Instrument

Physical performance was assessed using the Short Physical Performance Battery (SPPB). Anxiety and depression variables were assessed with the Hospital Anxiety and Depression Scale (HADS), sleep quality with the Pittsburgh Sleep Quality Index (PSQI), and stress level with the Perceived Stress Scale (PSS).

#### 2.5.1. Physical Performance

The SPPB was used to assess physical performance in older adults. It consists of three tests: gait speed, balance, and chair rise. It is useful in predicting several adverse health outcomes in this population. The SPPB is a reliable and valid tool for assessing physical function in older adults. However, it has limitations for its widespread use in specific populations, such as people with dementia, due to their limited ability to follow instructions [14].

#### 2.5.2. Anxiety and Depression

The Hospital Anxiety and Depression Scale (HADS) was used, a valid and frequently used instrument for assessing anxiety and depression in older adults. Although it may have certain diagnostic limitations in some cases, its use is highly recommended as a tool for assessing change over time, but not as a first-level diagnostic instrument [15].

#### 2.5.3. Perceived Stress

The Perceived Stress Score (PSS) was used to measure this variable. This test is used to measure perceived stress in different populations, including older adults. It measures how people perceive their stress level based on their daily experiences. The PSS is a valid and reliable scale, and its subscales provide insight into the relationship between stress and cognitive health [16,17,18].

#### 2.5.4. Sleep Quality

The Pittsburgh Sleep Quality Index (PSQI) was used to assess sleep quality. This is an important instrument for identifying sleep-related problems that could affect the quality of life in this population group. The PSQI measures sleep quality and patterns in older adults, identifying “good” and “poor” sleepers based on seven components: subjective sleep quality, sleep latency, sleep duration, habitual sleep efficiency, sleep disturbances, use of sleep medication, and daytime dysfunction [19,20].

##### Instrument Cut-Points and Validated Versions

Anxiety and depression were categorized using HADS standard cut-points (Normal 0–7, Borderline 8–10, Clinical 11–21 for each subscale). Poor sleep quality was defined as PSQI > 5. PSS-10 ranges 0–40 (higher = greater perceived stress). We used validated Spanish versions of HADS, PSQI, and PSS-10 appropriate for older adults, and all instruments were scored strictly according to their official manuals.

##### Administration and Measurement Considerations

The questionnaires were administered individually in a quiet room, free from any distractions or disturbances that could interfere with their completion.

Scales validated in Spanish were used, since 100% of the sample spoke that language. Furthermore, validation of the tools in Ecuadorian Spanish was not necessary, since the validated scales did not compromise comprehension of the information.

To ensure the reliability of the responses, assistive technologies were provided to those who required them, such as reading the questions aloud. It is emphasized that, under no circumstances, were answers or interpretations of the questions in the tools provided, thus maintaining the integrity of the self-assessment. Participants wore glasses or hearing aids as usual, without any disruption to the data collection process.

It is also important to note that the researchers who collected the data did not previously know the study participants and acted solely as external observers. In this way, expectation bias, also known as examiner bias, was minimized.

### 2.6. Data Collection

First, the directors of the eight nursing homes in the canton were located and contacted. The research protocol was presented to them, and the corresponding letter of interest was obtained.

Subsequently, potential participants were provided with a detailed explanation of the study at each institution, and their informed consent for voluntary participation was requested. Over a period of eight weeks, the two principal investigators, assisted by four assistants, applied the previously described instruments to assess all variables of interest. Finally, the data were verified, tabulated, and consolidated into an electronic database, ready for statistical analysis.

Sociodemographic data (age, gender) were collected through a simple interview. For the variables of interest (physical performance, anxiety and depression level, perceived stress level, and sleep quality), validated instruments were administered individually and sequentially in a quiet place to ensure participants’ comfort and concentration.

#### Data Quality and Bias Control

Procedures were standardized using an operating manual and checklists. Evaluators were trained and practiced the SPPB under supervision. Physical tests were performed under controlled conditions (same chair height and walking distance, calibrated stopwatch, standardized instructions) and preferably in the morning to minimize fatigue. The questionnaires (HADS, PSQI, PSS-10) were administered in a quiet environment, read aloud only when necessary, and without prompting responses; the use of glasses and hearing aids was permitted. Data quality was ensured with double-checking of loading and range, and logic controls.

### 2.7. Data Analyses

All analyses were performed in IBM SPSS Statistics v25. Two-sided tests with α = 0.05 were used.

#### 2.7.1. Descriptives and Bivariate Exploration

Physical performance (SPPB) and perceived stress (PSS-10) were summarized as mean ± SD and range; HADS-A/HADS-D and PSQI categories were presented as n (%). Continuous-variable distribution was examined with Shapiro–Wilk, and homogeneity of variances with Levene’s test when applicable. As exploratory descriptors, we reported rank-based bivariate correlations (Spearman’s ρ) between SPPB and mental-health measures, with exact *p*-values and conventional effect-size benchmarks (≈0.10 small, 0.30 medium, 0.50 large). Where groupwise descriptive contrasts were shown (e.g., HADS or PSQI categories), we reported medians [IQR] by category and used nonparametric tests (Kruskal–Wallis with ε^2^ = (H − k + 1)/(N − k) and Mann–Whitney U with r = |Z|/√N); these were considered descriptive/exploratory. Because heteroscedasticity was present in some contrasts, *p*-values are provided as descriptive/reference only; table footnotes make this explicit and indicate that primary inference relies on the single multivariable model with heteroskedasticity-robust 95% CIs. When multiple pairwise comparisons were conducted, Bonferroni adjustments were applied.

#### 2.7.2. Primary Analysis

The primary inferential model was a single multivariable linear regression (OLS) with SPPB as the dependent variable and HADS-A (anxiety), HADS-D (depression), PSS-10 (perceived stress), and PSQI (sleep quality; higher = worse) entered simultaneously as continuous predictors, adjusting for age (years) and sex (coded 1 = male, 2 = female). We report unstandardized coefficients (B) and two-sided *p*-values; inference (SEs and 95% CIs) was based on heteroskedasticity-consistent (Huber–White) estimates for all coefficients.

##### Model Diagnostics and Robustness Checks

Multicollinearity was assessed using variance inflation factors (VIFs), tolerance, and condition indices. Assumptions were inspected via standardized residuals vs. fitted values (to assess homoscedasticity) and normal P–P plots (residual normality). Influential observations were flagged using Cook’s distance > 4/n and centered leverage > 2 p/n (*p* = number of parameters including the intercept). As a sensitivity analysis, we re-estimated the OLS model excluding influential observations and compared effect directions and statistical significance.

To ensure robustness to potential violations of constant variance, we also estimated a generalized linear model with Normal distribution and identity link using robust (Huber–White) covariance; results are presented alongside the OLS estimates for comparison. In addition, acknowledging the bounded/ordinal nature of the SPPB scale (0–12), we fitted a proportional-odds (cumulative logit) model including the same predictors; we report the test of parallel lines, and treat these estimates as sensitivity results.

##### Multiplicity and Sample Used

Bonferroni corrections were applied only to preplanned pairwise post hoc contrasts; primary inference relied on the single multivariable model. All analyses used complete data on the analytic variables (*N* = 105).

## 3. Results

### 3.1. Population Characteristics

**Figure 1:**
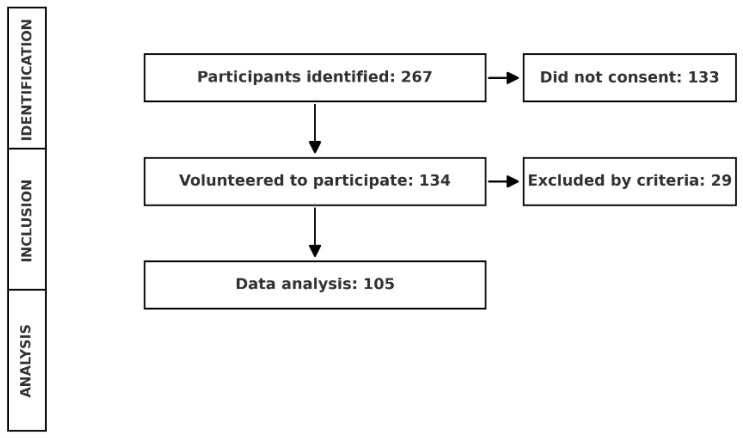
Participant selection and data analysis flowchart.

A total of 267 older adults were identified in the various geriatric nursing homes, of which 134 agreed to participate voluntarily. Following this, and after applying the selection criteria, the final population consisted of 105 older adults (Figure 1).

Table 1 describes the demographic characteristics of the sample, which consisted primarily of women (52%). The mean age is 80.78 years (SD ± 7.91), where the minimum age is 61 and the maximum age is 104. Regarding ethnicity, the majority identified themselves as mestizo (89.5%).

In the total sample (*N* = 105), the mean physical performance score (SPPB) was 6.94 ± 3.17 (range 0–12), while perceived stress averaged 16.73 ± 6.67 (range 0–36). The majority of participants showed poor physical condition (77.1%) and poor sleep quality (74.3%). Regarding mental health variables, normal levels predominated in both anxiety (58.1%) and depression (76.2%); the percentages of cases in the clinical problem range were 19.0% for anxiety and 11.4% for depression (Table 2).

### 3.2. Relationship Between Physical Performance and Mental Health

#### 3.2.1. Relationship Between Physical Performance and Anxiety

The overall analysis indicated significant differences in the SPPB score according to the anxiety level (*H*(2) = 9.55, *p* = 0.008). Post-hoc comparisons revealed that the group with anxiety in the clinical problem range obtained a significantly lower SPPB score than the normal group (7.41 ± 2.91 vs. 6.55 ± 3.69; *U* = 229, Z = −2.93, *p* = 0.003, *r* = 0.31). No differences were observed between the normal and doubtful groups (*p* = 0.183, *r* = 0.14) or between doubtful and clinical problem groups (*p* = 0.170, r = 0.27) after Bonferroni correction (α = 0.0167) (Table 3).

#### 3.2.2. Relationship Between Physical Performance and Depression

The SPPB score differed significantly across depression levels (*H*(2) = 9.55, *p* = 0.008, ε^2^ ≈ 0.07). The Normal group had the highest mean (7.46 ± 3.02), followed by the Doubtful group (6.00 ± 3.27) and the Clinical Problem group (4.50 ± 2.88). Post-hoc tests showed that only the Normal vs. Clinical Problem comparison was significant after Bonferroni correction (*U* = 229, *Z* = −2.93, *p* = 0.003, *r* = 0.31), indicating a medium effect; the differences between Normal and Doubtful (*p* = 0.183, *r* = 0.14) as well as between Doubtful and Clinical Problem (*p* = 0.170, *r* = 0.27) did not reach significance. These results suggest that depression in the clinical problem range is associated with lower physical performance, while the doubtful state does not differ from the normal range (Table 4).

#### 3.2.3. Relationship Between Physical Performance and Sleep Quality

Participants with good sleep quality achieved an average SPPB score of 8.81 ± 1.71, significantly higher than those with poor sleep quality (6.29 ± 3.30). The Mann–Whitney test confirmed the difference (*U* = 555.50, *Z* = −3.67, *p* < 0.001), with a medium effect size (*r* = 0.36), indicating that good sleep quality is associated with greater physical performance in this population (Table 5).

#### 3.2.4. Relationship Between Physical Performance and Perceived Stress

The mean physical performance score was 6.94 ± 3.17, while perceived stress averaged 16.73 ± 6.67 points. Spearman’s analysis revealed a weak negative association between the two variables (*ρ* = −0.20, *p* = 0.042), indicating that higher levels of stress were associated with lower physical performance in the older adults assessed (Table 6).

Bivariate correlations were retained as exploratory descriptors. Primary inference stems from the single multivariable model including HADS-D, HADS-A, PSS-10, and PSQI adjusted for age and sex.

#### 3.2.5. Adjusted Associations Between Mental-Health Measures and SPPB (Single Multivariable Model)

In the single multivariable model adjusted for age and sex, higher depressive symptoms (HADS-D) and poorer sleep quality (PSQI) were independently associated with lower SPPB scores, whereas anxiety (HADS-A), perceived stress (PSS-10), and sex were not; older age was also associated with lower SPPB. Using a generalized linear model with robust (Huber–White) standard errors, inferences were unchanged (Supplementary Table S1).

Results were also robust to the exclusion of influential observations defined as Cook’s D > 4/n or leverage > 2 p/n (*N* = 97); effect directions were preserved, with HADS-D and age remaining significant and the PSQI association attenuating to non-significance (Supplementary Table S2).

Multivariable linear regression of SPPB on anxiety (HADS-A), depression (HADS-D), perceived stress (PSS-10), and sleep quality (PSQI), adjusted for age and sex (*n* = 105). Unstandardized coefficients (B) represent the expected change in SPPB per 1-point increase in each predictor, holding other variables constant. Higher HADS-A/HADS-D/PSS-10 scores indicate greater symptom burden; higher PSQI indicates poorer sleep quality. Collinearity diagnostics did not indicate concern (VIF range 1.03–2.02; minimum tolerance 0.496). Model fit: adjusted *R*^2^ = 0.198; *F* (6, 98) = 5.280, *p* < 0.001. Sex was coded as 1 = male, 2 = female (Table 7).

Model fit indices and diagnostic statistics for the multivariable linear regression predicting SPPB from HADS-A, HADS-D, PSS-10, PSQI, age, and sex (*N* = 105). Reported metrics include R^2^, adjusted R^2^, standard error of the estimate, overall F test, variance inflation factors (VIFs), minimum tolerance, maximum condition index, maximum Cook’s distance, and maximum centered leverage. Residual diagnostics were inspected via normal P–P plots and standardized residuals vs. fitted values (ZRESID vs. ZPRED). Collinearity was acceptable (VIFs < 5; tolerance > 0.20), and residual patterns were broadly satisfactory. A small number of influential observations were identified (benchmark thresholds ≈ Cook’s D > 4/n and leverage > 2 p/n), so findings were corroborated with robustness checks as recommended (Table 8).

As a sensitivity analysis acknowledging the bounded/ordinal nature of SPPB, we fitted a proportional-odds (cumulative logit) model including HADS-D, HADS-A, PSS-10, PSQI, age, and sex. The model improved over the intercept-only model (*χ*^2^ (6) = 26.53, *p* < 0.001). The proportional-odds assumption was violated (Test of Parallel Lines *p* < 0.001), so results are presented as sensitivity only. Higher depressive symptoms and poorer sleep quality were associated with lower odds of being in higher SPPB categories, age was inversely associated, anxiety showed a positive adjusted association, and perceived stress was not associated (Supplementary Table S3).

## 4. Discussion

The present study partially confirms the hypothesis that higher physical performance is associated with better mental health, better sleep quality, and lower perceived stress among institutionalized older adults. While bivariate (only used for exploratory descriptive purposes) contrasts suggested associations of SPPB with anxiety, depression, sleep quality, and perceived stress, only the relationships with depression and sleep quality remained in adjusted analyses; anxiety and perceived stress attenuated after controlling for age and sex. Age showed a consistent inverse association with SPPB across models. Results were robust to different specifications and diagnostic checks: in a GLM with robust (Huber–White) covariance, inferences were unchanged (Supplementary Table S1); after excluding influential observations (Cook’s D > 4/n or leverage > 2 p/n), effect directions were preserved—depression and age remained significant—while the PSQI association attenuated to non-significance (Supplementary Table S2); and treating SPPB as ordinal yielded directionally consistent estimates (Supplementary Table S3), although the parallel-lines assumption was violated.

Lower SPPB scores were associated with clinical levels of both anxiety and depression in descriptive analyses, consistent with prior reports showing that poorer physical functioning is linked to greater vulnerability to mood symptoms in institutionalized older adults [21,22,23]. Low strength, slower gait speed, and impaired balance—SPPB components—have been implicated as correlates of anxious–depressive symptomatology and autonomy loss, potentially reinforcing inactivity and emotional deterioration [21]. In the multivariable model, the independent association was stronger and more consistent for depression, aligning with longitudinal evidence that reduced physical performance predicts subsequent depression [23] and with studies showing negative correlations between physical tests and depressive symptom scales [21,22].

By contrast, the adjusted association for anxiety did not remain globally significant in the original GLM framework and, when modeled simultaneously with depression and sleep, tended to display a positive coefficient—a pattern compatible with statistical suppression due to shared variance with depressive symptoms and sleep quality. This suggests that the portion of anxiety not overlapping with depression/sleep may relate differently to SPPB and should be interpreted cautiously. Overall, the adjusted pattern emphasizes the primacy of depressive symptoms for functional performance in this setting, while still acknowledging the clinical plausibility of anxiety as a co-occurring marker of vulnerability [21,22,23].

The observed association between better physical performance and good sleep quality is consistent with evidence from multiple studies reporting a direct relationship between sleep quality and physical performance [21,24,25,26,27]. For example, Eun et al. found that older women with good subjective sleep were more likely to have physical performance within the functional range [26]. In multicultural studies, such as that of Oliveira et al., it has been confirmed that both daytime sleepiness and poor sleep quality predict functional decline in older adults [24].

In our primary multivariable model, poorer PSQI (higher scores) was independently associated with lower SPPB, consistent with sleep’s role in physiological recovery and emotional regulation relevant to mobility. Robust-SE estimates confirmed this inference (S1). In the influence-sensitivity analysis (S2), the PSQI effect attenuated to non-significance after removing high-influence observations; the direction remained the same. Taken together, these findings support a clinically meaningful, but potentially heterogeneous, sleep–function link in institutional settings [25].

The negative correlation, although weak, between perceived stress and physical performance is consistent with studies that have documented that higher levels of stress are associated with reduced physical abilities and functional decline [28,29]. However, when adjusting for age and sex, the coefficient for stress did not reach significance, suggesting that part of the association observed in the bivariate analysis could be explained by age gradients and their interrelation with sleep and depression. This interrelation is consistent with emerging evidence suggesting that chronic stress in old age affects sleep quality and may contribute to deteriorating physical and mental health [24,26,30].

In long-term care settings, such as nursing homes, perceived stress can be influenced by psychosocial factors such as lack of autonomy, isolation, or perceived institutional burden. Periodic assessment can provide key information for comprehensive preventive interventions.

### 4.1. Clinical and Public Health Implications

These findings reinforce the need to integrate physical activity programs as part of standard care in nursing homes, not only to prevent falls and physical decline, but also as a therapeutic tool for emotional well-being.

Based on the adjusted analyses, screening and intervention priorities should prominently include depression and sleep quality, while also monitoring anxiety and stress. Interventions should include systematic assessment of variables such as anxiety, depression, sleep quality, and perceived stress, considering their impact on functionality.

Similarly, the results encourage the design of interdisciplinary programs where physical therapy, psychology, and medical staff work together. This is aligned with the proposal for older adult-centered care models that integrate physical and mental health as inseparable elements of well-being.

### 4.2. Future Lines of Research

This study opens new lines of research. On the one hand, it is necessary to promote longitudinal studies that allow for establishing the causal relationship between physical performance and mental health, as well as analyzing the mediating role of variables such as social support and perceived self-efficacy. Furthermore, it is recommended to incorporate complementary tools such as actigraphy to objectify sleep quality and stress biomarkers, which would reinforce the conclusions.

On the other hand, it is also important to evaluate combined interventions (exercise plus cognitive therapy or sleep management) and their impact on functional autonomy and quality of life.

Finally, it is also important to replicate this study in other geographical contexts and include non-institutionalized older adults, which would allow us to verify whether the residential environment modulates the identified associations.

### 4.3. Limitations

This research study had some limitations. On the one hand, the cross-sectional design prevents causal inference, so we can only discuss how variables were related without knowing their case mix. On the other hand, household convenience sampling can limit external validity and introduce selection bias. Similarly, allowing participants with moderate cognitive impairment and relying on self-administered scales (HADS, PSQI, PSS-10) can lead to measurement errors, despite their standardized administration. Furthermore, residual confounding is possible (particularly due to polypharmacy, multimorbidity burden, cognitive status strata above the exclusion threshold, and facility-level activity programming) as well as sedative/antidepressant use, functional dependence (ADL/IADL), and BMI, factors not fully reflected in our primary models. Because the participants in this study were institutionalized older adults, errors in interpreting the questionnaire questions are possible, as this population group is more likely to have attention, comprehension, and insight problems. Although this risk was mitigated by technical aids (such as reading the questionnaires aloud and using hearing aids or glasses, specific to each participant), it is not possible to eliminate this bias. Finally, environmental factors in institutions (such as staff, residents’ routines, and/or lighting and noise) may also be influential, as they influence both sleep and mobility, but were not measured.

## 5. Conclusions

This study provides evidence of an association between physical performance and mental-health constructs in institutionalized older adults. In a single multivariable model adjusted for age and sex, depression and sleep quality retained independent associations with physical performance assessed using the SPPB, whereas the bivariate relationships with anxiety and perceived stress attenuated and did not reach statistical significance. Age showed a consistent inverse association with physical performance across analyses. These findings were robust to alternative specifications—results were unchanged with robust (Huber–White) standard errors and were directionally preserved after excluding influential observations; treating SPPB as an ordinal outcome yielded consistent directions of effect.

Taken together, the results reinforce physical performance as a meaningful, integrative indicator of well-being in residential care and support interdisciplinary interventions that combine exercise with targeted management of depression and sleep problems, while monitoring anxiety and stress. Given the cross-sectional design, causal inference is precluded; however, the patterns observed provide a sound basis for prospective cohorts and multicomponent trials to clarify directionality, explore mechanisms, and refine prevention and treatment strategies in institutionalized older adults.

## Figures and Tables

**Table 1:** Sociodemographic characteristics of the participants (*N* = 105).

Characteristic	Indicator	Percentage
Gender	Male	47.6%
	Female	52.4%
Ethnicity	Mestizo	89.5%
	White	1.0%
	Indigenous	1.9%
	Afro-descendant	7.6%
**Age (quantitative)**	Mean ± SD: M = 80.78 SD ± 7.91	

**Table 2:** Descriptive statistics of main variables.

Variable (Type)	Descriptives (*N* = 105)
Physical performance (quantitative)	Mean ± SD (range): 6.94 ± 3.17 (0–12)
Physical condition (categorical)	Frequency (%): Good 24 (22.9%), Poor 81 (77.1%)
Anxiety (categorical)	Frequency (%): Normal 61 (58.1%), Uncertain 24 (22.9%), Clinical Problem 20 (19.0%)
Depression (categorical)	Frequency (%): Normal 80 (76.2%), Uncertain 13 (12.4%), Clinical Problem 12 (11.4%)
Sleep quality (categorical)	Frequency (%): Good 27 (25.7%), Poor 78 (74.3%)
Perceived stress (quantitative)	Mean ± *SD* (range): 16.73 ± 6.67 (0–36)

*Note.* For quantitative variables, the mean ± standard deviation and observed range are presented; for categorical variables, the absolute frequency and valid percentage of the total sample are presented.

**Table 3:** Global (Kruskal–Wallis) and post hoc (Mann–Whitney) comparison of the SPPB score between anxiety levels.

Comparison (*n*)	Mean ± SD (SPPB Score)	Test	Statistical/gl	*p*	Effect Size	Interpretation
Global: Normal 61, Doubtful 24, Problem 20	Normal 7.41 ± 2.91 Doubtful 6.08 ± 3.24 Problem 6.55 ± 3.69	Kruskal–Wallis	*H*(2) = 9.55	0.008	ε^2^ ≈ 0.07	Overall difference between levels
Normal vs. Clinical Problem	7.41 ± 2.91 vs. 6.55 ± 3.69	Mann–Whitney *U*	*U* = 229, *Z* = −2.93	0.003	*r* = 0.31	Normal > Problem
Normal vs. Doubtful	7.41 ± 2.91 vs. 6.08 ± 3.24	Mann–Whitney *U*	*U* = 400.5, *Z* = −1.33	0.183	*r* = 0.14	No difference
Doubtful vs. Clinical Problem	6.08 ± 3.24 vs. 6.55 ± 3.69	Mann–Whitney *U*	*U* = 53, *Z* = −1.37	0.170	*r* = 0.27	No difference

*Note.* Exploratory, descriptive contrasts: medians [IQR] for SPPB by anxiety categories were Normal = 7.0 [6,7,8,9] (*n* = 61), Doubtful = 6.0 [4,5,6,7,8] (*n* = 24), and Clinical problem = 7.0 [4,5,6,7,8,9,10] (*n* = 20). Mann–Whitney *p*-values (when reported) are shown for reference only; primary inference relies on the multivariable model with heteroskedasticity-robust 95% CIs (Table 7).

**Table 4:** Global (Kruskal–Wallis) and post-hoc (Mann–Whitney) comparison of the SPPB score between depression levels.

Comparison(*n*)	Mean ± SD (SPPB Score)	Test	Statistical/gl	*p*	Effect Size	Interpretation
Global: Normal 80, Doubtful 13, Problem 12	Normal 7.46 ± 3.02 Doubtful 6.00 ± 3.27 Problem 4.50 ± 2.88	Kruskal–Wallis	*H*(2) = 9.55	0.008	ε^2^ ≈ 0.07	Significant overall difference
Normal vs. Clinical problem	7.46 ± 3.02 vs. 4.50 ± 2.88	Mann–Whitney *U*	*U* = 229, *Z* = −2.93	0.003	*r* = 0.31	Normal > Problem
Normal vs. Doubtful	7.46 ± 3.02 vs. 6.00 ± 3.27	Mann–Whitney *U*	*U* = 400.50, *Z* = −1.33	0.183	*r* = 0.14	No difference
Doubtful vs. Clinical problem	6.00 ± 3.27 vs. 4.50 ± 2.88	Mann–Whitney *U*	*U* = 53, *Z* = −1.37	0.170	*r* = 0.27	No difference

*Note.* Exploratory, descriptive contrasts: medians [IQR] for SPPB by depression categories were Normal = 7.0 [6,7,8,9,10] (*n* = 80), Doubtful = 7.0 [4–8.5] (*n* = 13), and Clinical problem = 5.0 [2.25–6.75] (*n* = 12). Mann–Whitney *p*-values (when reported) are shown for reference only; primary inference relies on the multivariable model with heteroskedasticity-robust 95% CIs (Table 7).

**Table 5:** Comparison of SPPB score between sleep quality levels (Mann–Whitney U).

Comparison(*n*)	Mean ± SD (SPPB Score)	Test	Statistical/gl	*p*	*r*	Interpretation
Good sleep quality (27) vs. Poor sleep quality (78)	8.81 ± 1.71 vs. 6.29 ± 3.30	Mann–Whitney *U*	*U* = 555.50, *Z* = −3.67	<0.001	0.36	Good > Bad (medium effect)

*Note A.* Nonparametric Mann–Whitney *U* test (two-tailed, α = 0.05). The effect size was calculated as *r* = |Z|/√*N* (*N* = 105); reference values: small ≈ 0.10, medium ≈ 0.30, large ≈ 0.50. An *r* = 0.36 indicates a medium-sized effect. *Note B.* Exploratory, descriptive contrasts: medians [IQR] for SPPB by sleep quality were Good = 8.0 [7,8,9,10,11] (*n* = 27) and Poor = 6.0 [4,5,6,7,8,9] (*n* = 78). Mann–Whitney *p*-values (when reported) are shown for reference only; primary inference relies on the multivariable model with heteroskedasticity-robust 95% CIs (Table 7).

**Table 6:** Descriptives and correlation (Spearman) between physical performance (SPPB) and perceived stress.

**Descriptives**	***N***	**Mean ± *SD* (Points)**		
SPPB	105	6.94 ± 3.17		
Perceived stress	105	16.73 ± 6.67		
**Correlated variables**	***N***	***ρ*** **(Spearman)**	***p***	**Interpretation**
SPPB ↔ Perceived stress (0–40 pts)	105	−0.20	0.042	Weak, significant negative relationship

*Note.* A higher stress score is associated with lower physical performance; |ρ| ≈ 0.20 is considered a small effect.

**Table 7:** Multivariable linear regression predicting SPPB score (*n* = 105).

Predictor	*B*	SE	Std. Beta	95% CI for B (Robust)	*p*	VIF
HADS-A (Anxiety)	+0.224	0.094	0.298	0.038 to 0.410	0.019	2.018
HADS-D (Depression)	−0.230	0.085	−0.294	−0.398 to −0.061	0.008	1.534
PSQI (Sleep quality; higher = worse)	−0.187	0.082	−0.230	−0.351 to −0.024	0.025	1.322
PSS-10 (Perceived stress)	−0.062	0.056	−0.130	−0.173 to 0.050	0.275	1.821
Age (years)	−0.087	0.036	−0.218	−0.158 to −0.017	0.016	1.032
Sex (1 vs. 2)	−0.144	0.603	−0.023	−1.341 to 1.054	0.812	1.185

*Note.* Unstandardized coefficient. SE, 95% CIs and *p*-values are from a Generalized Linear Model (Normal distribution, Identity link) with a heteroskedasticity-consistent (Huber–White/HC) covariance estimator; results are robust to variance heterogeneity. Predictors were entered simultaneously. Reference category: Sex = female. (If shown, Std. Beta and VIF are from the corresponding OLS model and reported for comparability/multicollinearity).

**Table 8:** Model fit and diagnostics.

Statistic	Value
*R* ^2^	0.244
Adjusted *R*^2^	0.198
Standard error of the estimate	2.837
Model F (df = 6, 98)	5.280, *p* < 0.001
VIF range	1.03–2.02
Minimum tolerance	0.496
Maximum condition index	37.98 (no problematic variance concentration among predictors)
Maximum Cook’s distance	0.081
Maximum centered leverage	0.240
Residual normality (P–P plot)	Approximately linear
Homoscedasticity (ZRESID vs. ZPRED)	No clear funnel pattern

## Data Availability

For ethical restrictions, the raw data supporting the conclusions of this article will be made available by the authors on request.

## Supplementary Materials

The following supporting information can be downloaded at: https://www.mdpi.com/article/10.3390/healthcare13243306/s1. Table S1: Multivariable linear regression of SPPB with robust (Huber–White) standard errors. Table S2: OLS excluding influential observations. Table S3: Proportional-odds (cumulative logit) model for SPPB categories.

## Abbreviations

The following abbreviations are used in this manuscript:

SPPB	Short Physical Performance Battery
GAD-7	Generalized Anxiety Disorder-7
PHQ-9	Patient Health Questionnaire-9
PSQI	Pittsburgh Sleep Quality Index
PSS-10	Perceived Stress Scale—10 ítems
ADL	Activities of Daily Living
IADL	Instrumental Activities of Daily Living
BMI	Body Mass Index

## References

[1] Luo M.S., Chui E.W.T., Li L.W. (2020). The Longitudinal Associations between Physical Health and Mental Health among Older Adults. Aging Ment. Health.

[2] Reynolds C.F., Jeste D.V., Sachdev P.S., Blazer D.G. (2022). Mental health care for older adults: Recent advances and new directions in clinical practice and research. World Psychiatry.

[3] Informe Mundial Sobre El Envejecimiento y La Salud. https://www.who.int/es/publications/i/item/9789241565042.

[4] Visualizador de Proyecciones Poblacionales. https://www.ecuadorencifras.gob.ec/proyecciones-poblacionales/.

[5] Monteiro-Junior R.S., Figueiredo L.F.d.S., Maciel-Pinheiro P.d.T., Abud E.L.R., Engedal K., Barca M.L., Nascimento O.J., Laks J., Deslandes A.C. (2017). Virtual Reality-Based Physical Exercise With Exergames (PhysEx) Improves Mental and Physical Health of Institutionalized Older Adults. J. Am. Med. Dir. Assoc..

[6] Wong M.Y.C., Ou K.L., Chung P.K., Chui K.Y.K., Zhang C.Q. (2022). The relationship between physical activity, physical health, and mental health among older Chinese adults: A scoping review. Front. Public Health.

[7] Velaithan V., Tan M.M., Yu T.F., Liem A., Teh P.L., Su T.T. (2024). The Association of Self-Perception of Aging and Quality of Life in Older Adults: A Systematic Review. Gerontologist.

[8] McPhee J.S., French D.P., Jackson D., Nazroo J., Pendleton N., Degens H. (2016). Physical activity in older age: Perspectives for healthy ageing and frailty. Biogerontology.

[9] Keadle S.K., McKinnon R., Graubard B.I., Troiano R.P. (2016). Prevalence and trends in physical activity among older adults in the United States: A comparison across three national surveys. Prev. Med..

[10] Guralnik J.M., Ferrucci L., Simonsick E.M., Salive M.E., Wallace R.B. (1995). Lower-extremity function in persons over the age of 70 years as a predictor of subsequent disability. N. Engl. J. Med..

[11] Stubbs B., Vancampfort D., Smith L., Rosenbaum S., Schuch F., Firth J. (2018). Physical activity and mental health. Lancet Psychiatry.

[12] Envejecimiento y Salud. https://www.who.int/es/news-room/fact-sheets/detail/ageing-and-health.

[13] Freedman V.A., Martin L.G., Schoeni R.F. (2002). Recent trends in disability and functioning among older adults in the United States: A systematic review. JAMA.

[14] Kameniar K., Mackintosh S., Van Kessel G., Kumar S. (2024). The Psychometric Properties of the Short Physical Performance Battery to Assess Physical Performance in Older Adults: A Systematic Review. J. Geriatr. Phys. Ther..

[15] Roberts M.H., Fletcher R.B., Merrick P.L. (2014). The validity and clinical utility of the hospital anxiety and depression scale (HADS) with older adult New Zealanders. Int. Psychogeriatr..

[16] Jiang J.M., Seng E.K., Zimmerman M.E., Sliwinski M., Kim M., Lipton R.B. (2017). Evaluation of the Reliability, Validity, and Predictive Validity of the Subscales of the Perceived Stress Scale in Older Adults. J. Alzheimer’s Dis..

[17] Jiang J.M., Seng E.K., Zimmerman M.E., Kim M., Lipton R.B. (2017). Positively worded subscale score of the Perceived Stress Scale is associated with cognitive domain function. J. Behav. Brain Sci..

[18] Yılmaz Koğar E., Koğar H. (2024). A systematic review and meta-analytic confirmatory factor analysis of the perceived stress scale (PSS-10 and PSS-14). Stress Health.

[19] Smyth C. (1999). The Pittsburgh Sleep Quality Index (PSQI). J. Gerontol. Nurs..

[20] Wang L., Saito T., Yokote T., Chen C., Yatsugi H., Liu X., Kishimoto H. (2025). Associations between sleep duration and quality and physical frailty in community-dwelling older adults: A cross-sectional study. Sci. Rep..

[21] Yousef A.M., Adly N.N., Elbedewy R.M.S., Elsorady K.E. (2025). Indicators of Low Handgrip Strength and its Association with Poor Sleep Quality among Community-dwelling Older Adults: A Cross-Sectional Study in Egypt. Eur. J. Geriatr. Gerontol..

[22] Payne M.E., Porter Starr K.N., Orenduff M., Mulder H.S., McDonald S.R., Spira A.P., Pieper C.F., Bales C.W. (2018). Quality of Life and Mental Health in Older Adults with Obesity and Frailty: Associations with a Weight Loss Intervention. J. Nutr. Health Aging.

[23] Veronese N., Stubbs B., Trevisan C., Bolzetta F., De Rui M., Solmi M., Sartori L., Musacchio E., Zambon S., Perissinotto E. (2017). Poor Physical Performance Predicts Future Onset of Depression in Elderly People: Progetto Veneto Anziani Longitudinal Study. Phys. Ther..

[24] Oliveira R.L., Freitas R.L., Duarte Y.A.O., Santos J.L.F., de Andrade F.B. (2024). Longitudinal association of sleep quality with physical performance measures: SABE cohort study, Brazil. Public Health.

[25] Berkley A.S., Carter P.A., Yoder L.H., Acton G., Holahan C.K. (2020). The effects of insomnia on older adults’ quality of life and daily functioning: A mixed-methods study. Geriatr. Nurs..

[26] Eun K.J. (2013). Association of sleep quality with physical performance among Korean aging people. Eur. Geriatr. Med..

[27] Denison H.J., Jameson K.A., Sayer A.A., Patel H.P., Edwards M.H., Arora T., Dennison E.M., Cooper C., Baird J. (2021). Poor sleep quality and physical performance in older adults. Sleep Health.

[28] Suh S.R., Hong H.S. (2001). Stress, immune cells, physical health status and depression of elderly. J. Korean Biol. Nurs. Sci..

[29] Johnson R. (2018). Predictors of Sleep Quality: Depression, Anxiety, and Sleep Self-Efficacy. Bachelor’s Thesis.

[30] Chen W.C., Chen S.J., Zhong B.L. (2022). Sense of Alienation and Its Associations With Depressive Symptoms and Poor Sleep Quality in Older Adults Who Experienced the Lockdown in Wuhan, China, During the COVID-19 Pandemic. J. Geriatr. Psychiatry Neurol..

