# Supplementary material for: Physical Performance and Mental Health in Institutionalized Older Adults: A Multicenter, Cross-Sectional Observational Study
Source: Healthcare (Basel). 2025 Dec 17;13(24):3306. doi: 10.3390/healthcare13243306 (PMC12732505; doi:10.3390/healthcare13243306)
Supplement: Supplementary file 1 [file healthcare-13-03306-s001.zip › healthcare-3933878-supplementary.pdf]

Supplementary Table S1. Multivariable linear regression of SPPB with **robust (Huber–White) standard errors** (GLM, Normal distribution, identity link), including HADS-A, HADS-D, PSS-10, and PSQI as continuous predictors, adjusted for age and sex (n = 105). Unstandardized coefficients (B) represent the change in SPPB per 1-unit increase in each predictor. Robust standard errors and Wald 95% CIs are shown. Inferences were unchanged relative to the primary OLS model.

Tabla 1 Multivariable linear model with robust (Huber–White) standard errors

Predictor	B	Robust SE	95% CI for B	Wald χ^2	p
**Intercept**	16.367	3.046	10.396 to 22.338	28.866	<.001
**Sex (1 vs 2)**	0.144	0.570	−0.974 to 1.261	0.063	.801
**HADS-A (Anxiety)**	**+0.224**	0.081	**0.065 to 0.382**	7.622	**.006**
**HADS-D (Depression)**	**−0.230**	0.076	**−0.378 to −0.082**	9.250	**.002**
**PSQI (higher=worse)**	**−0.187**	0.086	**−0.356 to −0.019**	4.758	**.029**
**PSS-10 (Perceived stress)**	−0.062	0.053	−0.166 to 0.042	1.355	.244
**Age (years)**	**−0.087**	0.037	**−0.159 to −0.016**	5.728	**.017**

Notes. GLM with Normal distribution and identity link; **robust (Huber–White) covariance**. n=105. B = unstandardized coefficient (change in SPPB per one-unit increase in the predictor).

In an influence sensitivity analysis excluding observations with Cook's D > 4/n or leverage > 2p/n (n=97), effect directions were preserved. The associations for depressive symptoms and age remained significant, whereas the PSQI association attenuated and was no longer statistically significant (Supplementary Table S2).

Table 2 OLS excluding influential observations

Predictor	B	SE	95% CI for B	p
**HADS-A (Anxiety)**	**+0.267**	0.092	0.084 to 0.449	**.005**
**HADS-D (Depression)**	**−0.261**	0.090	−0.440 to −0.083	**.005**
**PSQI (higher = worse)**	−0.118	0.087	−0.290 to 0.054	.175
**PSS-10 (Perceived stress)**	−0.108	0.058	−0.223 to 0.007	.065
**Age (years)**	**−0.077**	0.034	−0.145 to −0.009	**.027**
**Sex (1 vs 2)**	−0.414	0.573	−1.552 to 0.725	.472

Notes. OLS model after excluding observations with **Cook's D > 0.038** or **leverage > 0.133** (n=97). R^2=0.261; adjusted R^2=0.212; $F(6,90)$=5.305, p<.001. VIF range 1.19–1.99.

Proportional-odds (cumulative logit) model treating SPPB as an ordinal outcome. Predictors include HADS-A (anxiety), HADS-D (depression), PSS-10 (perceived stress), PSQI (sleep quality; higher = worse), age (years), and sex (coded 1 = male, 0 = female). Values are odds ratios (OR) per 1-unit increase with 95% Wald confidence intervals and p values. OR < 1 indicates lower odds of being in a higher SPPB category (i.e., poorer physical performance). Model fitting: $\chi^2(6) = 26.53$, $p < .001$ versus intercept-only. The proportional-odds assumption was violated (Test of Parallel Lines $p < .001$), so these results are presented as a sensitivity analysis.

Table 3 Proportional-odds (cumulative logit) model for SPPB categories.

**Predictor**	**OR per 1-unit**	**95% CI**	**p**
**HADS-A (Anxiety)**	**1.145**	1.020–1.285	**.022**
**HADS-D (Depression)**	**0.869**	0.783–0.967	**.009**
**PSQI (higher=worse)**	**0.899**	0.813–0.994	**.039**
**PSS-10 (Perceived stress)**	0.969	0.905–1.038	.367
**Age (years)**	**0.947**	0.907–0.990	**.017**
**Sex (1 vs 2)**	1.156	0.558–2.396	.695

Notes. Link function: logit. “Model fitting information”: $\chi^2(6)=26.53$, $p<.001$. “Test of parallel lines”: $p<.001$ (assumption violated; results used as sensitivity)
